# Secretory leukoprotease inhibitor is required for efficient quercetin-mediated suppression of TNFα secretion

**DOI:** 10.18632/oncotarget.12415

**Published:** 2016-10-03

**Authors:** Stefania De Santis, Dale Kunde, Grazia Serino, Vanessa Galleggiante, Maria Lucia Caruso, Mauro Mastronardi, Elisabetta Cavalcanti, Nicole Ranson, Aldo Pinto, Pietro Campiglia, Angelo Santino, Rajaraman Eri, Marcello Chieppa

**Affiliations:** ^1^ IRCCS “de Bellis”, Laboratory of Experimental Immunopathology, Castellana Grotte (BA), Italy; ^2^ Institute of Sciences of Food Production, C.N.R. Unit of Lecce, Lecce, Italy; ^3^ Mucosal Biology, School of Human Life Sciences, University of Tasmania, Launceston, TAS, Australia; ^4^ Department of Pharmacy, School of Pharmacy, University of Salerno, Fisciano (SA), Italy; ^5^ IRCCS “de Bellis”, Department of Pathology, Castellana Grotte (BA), Italy; ^6^ IRCCS “de Bellis”, Department of Gastroenterology, Castellana Grotte (BA), Italy; ^7^ European Biomedical Research Institute of Salerno (EBRIS), Salerno, Italy

**Keywords:** polyphenols, dendritic cells, inflammation, SLPI, nutrition

## Abstract

Dendritic cells (DCs) are professional antigen presenting cells (APCs) that in response to microbial infections generate long-lasting adaptive immune response. Following microbial uptake, DCs undergo a cascade of cellular differentiation that ultimately leads to “mature” DCs. Mature DCs produce a variety of inflammatory cytokines, including tumor necrosis factor-α (TNFα) a key cytokine for the inflammatory cascade. In numerous studies, polyphenols, including quercetin, demonstrated their ability to suppress TNFα secretion and protect from the onset of chronic inflammatory disorders. We show that murine bone marrow derived DCs express Slpi following quercetin exposure. Slpi is known to suppress LPS mediated NFκB activation, thus, it was hypothesized that its expression could be the key step for polyphenol induced inflammatory suppression. Slpi-KO DCs poorly respond to quercetin administration failing to reduce TNFα secretion in response to quercetin exposure. Supernatant from quercetin exposed DCs could also reduce LPS-mediated TNFα secretion by unrelated DCs, but this property is lost using an anti-Slpi antibody. *In vivo*, oral administration of quercetin is able to induce Slpi expression. Human biopsies from inflamed tract of the intestine reveal the presence of numerous SLPI^+^ cells and the expression level could be further increased by quercetin administration. We propose that quercetin induces Slpi expression that in turn reduces the inflammatory response. Our data encourages the development of nutritional strategies to improve the efficiency of current therapies for intestinal chronic inflammatory syndrome and reduce the risks of colorectal cancer development.

## INTRODUCTION

Plant polyphenols represent one of the largest and most ubiquitous groups of secondary metabolites that are an integral part of the human diet [1, 2]. These compounds are characterized by the presence of one or more phenol rings and two or more hydroxyl groups linked directly to the aromatic rings [3] and have been associated with anti-oxidant, anti-microbial, anti-proliferative and anti-inflammatory properties [4, 5]. Despite evidence for the biological effects of these phytonutrients being reported, knowledge of the underlying molecular mechanisms activated upon polyphenols treatment remain poorly understood [6–10]. Polyphenols can mediate NF-kBp65 subunit translocation into the nucleus [10, 11] significantly modulating inflammatory cytokine secretion and we have recently reported that the inhibition of I kappa B kinase (IKK) phosphorylation is a crucial step in the cascade of events that starts with polyphenols exposure [12]. In addition, we described that the two polyphenols quercetin and piperine, suppressed endotoxic lipopolysaccharide (LPS) mediated bone marrow dendritic cells (BMDCs) activation [13]. We demonstrated that DCs exposed to quercetin and piperine fail to release inflammatory cytokines, to present antigens and to switch the chemokine receptors repertoire decreasing their ability to promote Th1 CD4^+^ polarization.

The secretory leukocyte protease inhibitor (Slpi) is a member of the innate immunity associated protein family which is secreted by several cell types including DCs, neutrophils and macrophages [14]. Slpi's main function appears to be tissue protection against the deleterious consequences of prolonged inflammation by suppressing an excessive inflammatory response [15, 16]. At the same time Slpi is a potent antimicrobial factor mainly produced in the mucosal tissues [17, 18] antagonizing LPS induced signaling and inflammatory cytokines secretion [14, 19]. Recently it has been described as a potent inflammatory inhibitory protein able to antagonize LPS induced activation of NFκB [14, 20]. Evidence of the importance of Slpi in inflammatory suppression and tissue repair has come from thymic stromal lymphopoietin (Tslp) knockout (KO) mice that have reduced Slpi expression [21]. These mice demonstrate a similar development of colitis as wild-type mice when exposed to dextran sulfate sodium (DSS), however have a higher mortality rate due to a reduced healing ability. This effect can be reversed in vivo with administration of soluble Slpi [21]. Recently, Slpi expressing DCs have been described in the cervical lymph nodes (LN) suggesting that its expression could also be crucial to modulate the mucosal DC activation in gut inflammation [22]. These data strongly suggest that Slpi can play a major role in the modulation of DCs in gut inflammation thus suggesting that therapeutic strategies promoting Slpi expression may be beneficial.

Inflammatory bowel diseases, which include Crohn's disease (CD) and ulcerative colitis (UC), are a growing global health problem. Incidence and prevalence of both CD and UC has traditionally been highest in the industrialized Western nations, however, in recent years, incidence has also been rapidly increasing in developing countries [23, 24]. Currently, treatment for IBD is symptomatic, therefore, patients with IBD must endure chronic, relapsing illness for the remainder of their lives. However, a reasonably successful but costly therapeutic target in IBD is the use of TNFα inhibitors, including Infliximab and similar agents, to reduce the effect of the TNFα mediated pro-inflammatory cascade associated with chronic inflammation. A significant complication associated with IBD, particularly UC is the development of colorectal cancer (CRC), which in recent years has been reported to be increasing in younger population [25]. The development of CRC is closely associated with chronic inflammation and raised TNFα secretion [26] leading to therapeutic strategies aimed at minimizing chronic inflammation in these patients through TNFα inhibitors, ultimately reducing the risk of its CRC [27, 28]. There has been recent interest in the use of natural or dietary based therapeutic strategies, particularly plant based polyphenols to reduce inflammatory responses in UC and its progression to CRC by modulating early inflammation in these conditions [12, 29, 30].

In the present study we report that quercetin up-regulates Slpi expression in DCs while playing a central role in reducing TNFα secretion. Importantly, our data shows that the absence of Slpi reduces the efficiency of DC response to polyphenols providing a rationale for developing polyphenol enriched diets to protect from TNFα mediated mucosal inflammatory responses.

## RESULTS

### Quercetin induces Slpi expression in LPS-activated BMDCs

**Figure 1:**
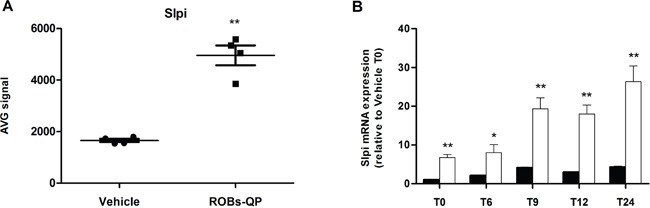
Quercetin induces Slpi expression in LPS-activated BMDCs **A.** Slpi expression from the microarray data of BMDCs exposed to vehicle or ROBs-QP at day 5 and 7 and treated with 1 μg/mL of LPS for 6 hours (n=4, **P<0.01). **B.** Time course mRNA expression of Slpi mRNA measured by qPCR of BMDCs exposed to vehicle (black bars) or ROBs-QP (white bars) at day 5 and 7 and treated with 1 μg/mL of LPS. Fold change are expressed relative to vehicle at time 0. mRNA was extracted at indicated time points and bars represent the mean ± SEM of 3 independent experiments. (*P<0.05, **P<0.01).

A whole-genome microarray analysis performed on mRNA extracted from Quercetin and Piperine Reconstituted Oil Bodies (ROBs-QP) treated BMDCs at 6 hours post LPS exposure identified a number of genes that were significantly modified by polyphenols exposure as previously described [13]. As expected, most of the transcripts differentially expressed by polyphenol exposure were those of pro-inflammatory genes, which were significantly down-regulated consistent with known anti-inflammatory activity of the polyphenols. We identified secretory leukocyte protease inhibitor (Slpi) among the transcripts differently up-regulated in polyphenol exposed BMDCs following LPS stimulation (2.7-fold, P<0.01; Figure 1A).

The microarray data was confirmed by qPCR where Slpi expression was significantly up-regulated in the ROBs-QP treated BMDCs reaching a 26-fold (P<0.01) increase at 24h post LPS activation compared to a 4-fold increase in vehicle treated BMDCs. Significantly, Slpi expression was increased in ROBs-QP treated BMDCs even before LPS activation (7-fold induction) (Figure 1B). Similar Slpi expression profiles were obtained using 25 μM of synthetic quercetin (data not shown). For this reason, we chose to study the effect of a single polyphenol administration and used synthetic quercetin for the remainder of the study.

### Quercetin reduces TNFα secretion by up-regulating Slpi

**Figure 2:**
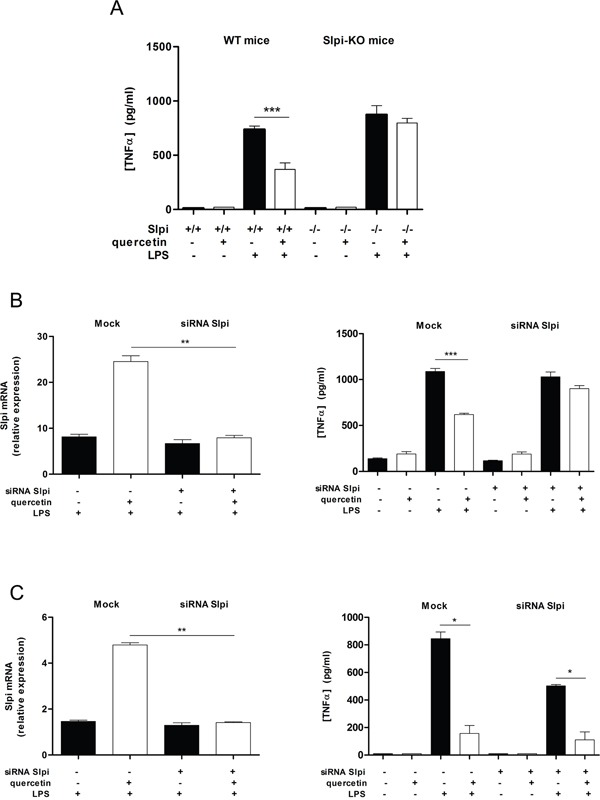
Quercetin reduces TNFα secretion by up-regulating Slpi **A.** BMDCs were cultured from WT and Slpi-KO mice and treated with quercetin at day 5 and 7. BMDCs cultures were exposed to 1 μg/mL of LPS and the secretion of TNFα was determined by ELISA after 24 hours. Bars represent mean cytokine concentration ± SEM (n=4) for BMDCs from WT or Slpi-KO mice treated with and without quercetin (black and white bars, respectively, ***P<0.001). **B.** BMDCs were transfected with siRNA for Slpi and Lipofectamine® 3000 reagent (mock) at day 4, before the administration of quercetin. qPCR for Slpi demonstrated a good efficiency for siRNA transfection (left). Bars represent a mean fold change ± SEM (n=3) between LPS stimulated BMDCs +/− quercetin relative to LPS unstimulated cells +/− quercetin, respectively. **P<0.01. The secretion of TNFα was determined 24 hours after LPS stimulation by ELISA (right). Bars represent mean cytokine concentration ± SEM (n=3) for DCs treated with and without quercetin (black and white bars, respectively). ***P<0.001. **C.** BMDCs were treated with quercetin on day 5 and transfected with siRNA for Slpi at day 6 followed by quercetin administration at day 7 and a subsequent exposure to LPS on day 8. A good reduction in Slpi expression was observed by qPCR analysis (left). Bars represent a mean fold change ± SEM (n=3) between LPS stimulated BMDCs +/− quercetin relative to LPS unstimulated cells +/− quercetin, respectively. **P<0.01. The secretion of TNFα was determined 24 hours after LPS stimulation by ELISA (right). Bars represent mean cytokine concentration ± SEM (n=3) for DCs treated with and without quercetin (black and white bars, respectively). *P<0.05.

BMDCs isolated from WT mice and treated with quercetin showed a significant reduction in secretion of TNFα upon activation with LPS compared to non-treated BMDCs (50% suppression, P<0.01), while BMDCs from Slpi-KO mice treated in the same way failed to reduce TNFα secretion (Figure 2A).

To further elucidate whether the observed difference in TNFα secretion in quercetin treated BMDCs from WT and Slpi-KO mice was indeed Slpi dependent we used siRNA to knockdown Slpi in WT BMDCs and assessed the TNFα secretion profile. siRNA knockdown of Slpi in BMDCs from WT mice (at day 4 of culture) also demonstrated a failure to reduce TNFα secretion after quercetin treatment and LPS activation (Figure 2B). In contrast, BMDCs from WT mice that had Slpi expression knocked-down after quercetin treatment (at day 6 of culture) suppressed TNFα secretion after exposure to LPS similar to mock siRNA treated BMDCs (Figure 2C).

### Extracellular Slpi suppresses TNFα secretion

**Figure 3:**
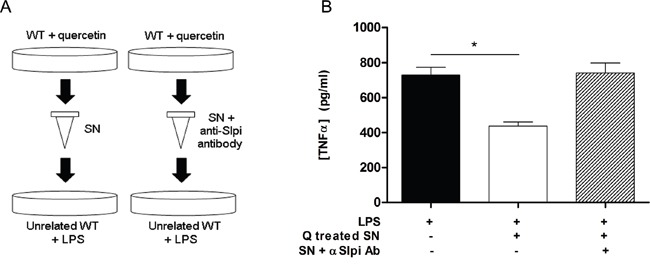
Extracellular Slpi suppresses TNFα secretion **A.** Supernatant collected from WT BMDCs culture stimulated at day 5 and day 7 with quercetin was added 1:1 to an unrelated BMDCs culture (left). After 1 hour the recipient BMDCs were stimulated with 1μg/ml of LPS for 24 hours and the amount of TNFα was tested by ELISA relative to SN untreated BMDCs (panel B, white bar and black bar, respectively). *P<0.05. **B.** To discriminate the contribution of the endogenous and the extracellular form of Slpi, the SN collected from WT BMDCs plus quercetin was treated with an anti-Slpi antibody (BAF1735, R&D, panel A, right). Supernatant incubated with the antibody was treated with LPS as before and tested by ELISA for TNFα secretion (panel B, stripped bar). Bars represent mean cytokine concentration ± SEM (n=3) for BMDCs from WT mice.

We measured TNFα secretion from LPS-activated WT BMDCs that had been treated with culture medium from previously quercetin-treated WT BMDCs (Figure 3A). The secretion of TNFα was significantly reduced in BMDCs exposed to medium taken from quercetin-treated WT-BMDCs after LPS activation compared to vehicle (59% suppression, P<0.001; Figure 3B). Pretreatment of the media from quercetin treated WT-BMDCs supernatant with a blocking anti-Slpi for 2 hours increased the secretion of TNFα after LPS activation to levels seen in vehicle treated BMDCs (Figure 3A, 3B).

### Orally administered quercetin induces Slpi expression in murine colon

**Figure 4:**
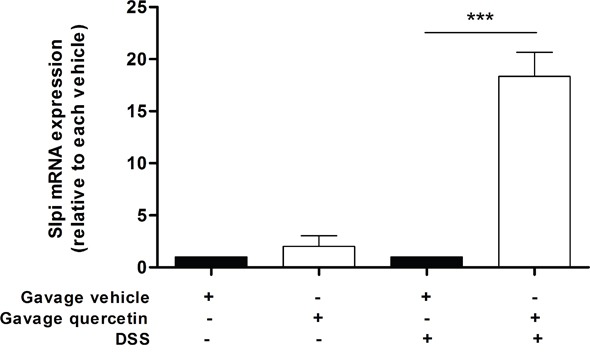
Orally administered quercetin induces Slpi expression in murine colon Gavage of quercetin or vehicle was administered at day 0, 3, 5 and 7. Starting from day 4, 2%DSS was administered for 3 days in drinking water. At day 7 mice were sacrificed, the colon collected and the mRNA extracted. Slpi expression was measured by qPCR in the colon of vehicle (black bars) and quercetin (white bars) treated mice exposed or not to DSS. Bars represent mean expression ± SEM (n=3) for each treatment. ***P<0.001.

To extend our findings of quercetin mediated increase in Slpi expression we investigated whether oral administration of quercetin would elicit similar effects on inflamed murine colon. WT mice treated with quercetin but not challenged with 7 days of 2% DSS did not show a significant increase in the Slpi mRNA expression (Figure 4). However, colonic tissue from mice treated with quercetin followed by 7 days of 2% DSS challenge shows a significant increase in Slpi mRNA expression (15-fold increase, P<0.001) compared to DSS untreated control mice (Figure 4).

### SLPI is expressed in human inflamed intestinal tract and further induced following quercetin exposure

**Figure 5:**
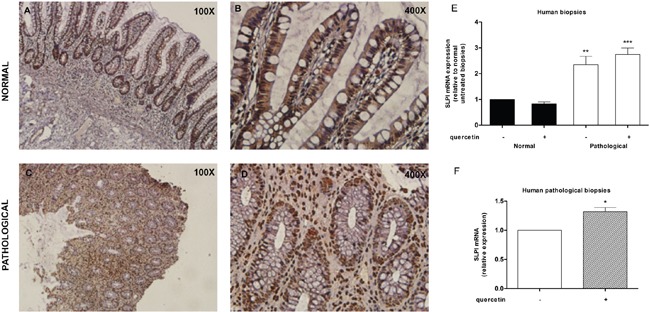
SLPI is expressed in human inflamed intestinal tract and further induced following quercetin exposure **A-D.** Representative immunohistochemistry images for SLPI expression in normal (panels A and B) and inflamed (panels C and D) human intestinal biopsies. FFPE sections were prepared at 20 μm thickness, stained for Slpi and visualized using BioCare polymer IHC kit. **E.** SLPI expression was measured on human normal and inflamed areas by qPCR relative to untreated non-inflamed biopsies. Bars represent mean ± SEM (n=7). **P<0.01; ***P<0.001. **F.** qPCR on human inflamed tissues treated with quercetin relative to inflamed untreated ones. Bars represent mean ± SEM (n=7). *P<0.05.

We investigated the expression of SLPI and the response to quercetin treatment in human inflammatory bowel disease. Immunohistochemistry of SLPI in biopsy tissues from UC patients (Figure 5, panels A-D) revealed consistent SLPI reactivity primarily in the epithelial layer of non-inflamed tissue while numerous SLPI^+^ infiltrating cells can be seen in the inflamed tissue. In the epithelial monolayer positivity was not altered. SLPI mRNA expression was significantly increased in active colitis biopsy tissue and quercetin treated active colitis tissue compared to quiescent region biopsy tissue (2.3-fold, P<0.01 and 2.7-fold, P<0.001, respectively), while quiescent tissues did not show significant variations (Figure 5E). Remarkably, quercetin treatment significantly increased SLPI expression compared to vehicle treated biopsies from active inflammation colonic tissues (1.2-fold, P<0.05; Figure 5F).

## DISCUSSION

In this study we demonstrate that quercetin up-regulates Slpi and this up-regulation is central in suppressing TNFα in response to LPS activation of immune cells, primarily DCs. Slpi is a member of the innate immunity associated protein family secreted by several cell types including DCs, neutrophils and macrophages. The functions of Slpi are many fold, ranging from protease inhibition, antimicrobial factor, suppression of pro-inflammatory cytokine expression and enhancement of tissue repair mechanisms. We propose for the first time that the well documented anti-inflammatory actions of quercetin are mediated through the up-regulation of Slpi, with downstream TNFα suppression.

Quercetin significantly up-regulated Slpi expression in un-activated BMDCs and greatly enhanced up-regulation of Slpi following LPS activation. DCs are being identified as central mediators of inflammation in the gut through cytokine release and controlling gut responses to microbiota [31]. Our data indicates that the up-regulation of Slpi, a protective mediator in DCs, may provide a mechanism to reduce the inflammatory responses. The ability of quercetin to up-regulate Slpi expression in DCs provides mechanistic insights into the previously reported benefits of polyphenols in colitis. It has been previously demonstrated that Slpi can suppress LPS-induced TNFα transcription [32] and in line with this observation, we have shown that quercetin pre-treatment significantly suppressed TNFα secretion. However, in the absence of Slpi, either in Slpi-KO mice or via siRNA knockdown, this suppression was ablated. These data suggest that the quercetin-induced TNFα suppression requires Slpi. Importantly, quercetin treated BMDCs promote Slpi mRNA expression even before LPS. Our data demonstrate that Slpi expression is a crucial checkpoint in the quercetin mediated anti-inflammatory response. Furthermore, we demonstrate that the quercetin mediated suppression of TNFα secretion is active in the presence of the secreted form of Slpi and this anti-inflammatory effect could be ablated by neutralizing soluble Slpi.

We have shown that inflamed colonic tissue expresses Slpi, probably in an anti-inflammatory protective response. However, its expression could be further up-regulated by polyphenols present in the lumen, potentially enhancing Slpi mediated protective and tissue repair mechanisms [21, 33]. We have previously shown that administration of high doses of polyphenols, including quercetin, reduce the DSS mediated acute intestinal inflammation [13]; a finding that data from the present study suggests is through the up-regulation of Slpi expression. We hypothesize that under homeostatic conditions, polyphenols exposure contributes to boost DCs production of Slpi preventing their ability to respond to LPS even in presence of mucosal insults causing intestinal barrier loss.

In human biopsies, the increase in the SLPI expression in inflamed tissue appears to be related to an increase in SLPI^+^ cells in the lamina propria. The observed expression, was further increased by quercetin exposure. Although the tissue was already inflamed and the exposure time was just 4 hours, we could detect an increase in the SLPI expression level. SLPI expression was not detectable in non-inflamed areas, but this observation may be explained by the lower ratio of immune cells present in the intestine in homeostatic condition.

In conclusion, this study indicates that quercetin exposure promotes Slpi expression in DCs as well as colonic tissue of UC patients thus favoring tissue repair and blocking inflammatory cytokine secretion. Our data suggests that quercetin (and possibly other polyphenols) administration may be effective in modulating the TNFα mediated pro-inflammatory cascade and help to generate tolerogenic Slpi^+^ DCs able to protect from chronic intestinal inflammation. These anti-inflammatory effects of polyphenols may provide natural dietary mechanisms to modulate inflammation and reduce the risk of chronic inflammation developing to colorectal cancer.

## MATERIALS AND METHODS

### Mice

*Ethics Statement*: investigation has been conducted in accordance with the ethical standards and according to the Declaration of Helsinki and according to national and international guidelines and has been approved by the authors' institutional review board.

6- to 8-week-old male mice were purchased from Jackson Laboratories: Wild-type C57BL/6 (Stock No: 000664; weight: approximately 20gr), B6;129-Slpi^tm1Smw^/J (Stock No: 010926; weight: approximately 20 g). All animal experiments were carried out in accordance with Directive 86/609 EEC enforced by Italian D.L. n. 116 1992, and approved by the official RBM veterinarian, as well as the Australian 0013329 & 130702 (UTAS Animal Ethics Committee). Animals were sacrificed if found in severe clinical condition in order to avoid undue suffering.

### Generation and culture of murine DCs

DCs were harvested from murine bone marrow (BM). Briefly, BMs from the tibiae and femurs of 6- to 8-week-old male C57BL/6 and Slpi-KO mice were flushed with 0.5mM EDTA (Thermo Fisher Scientific, MA, USA), and depleted of red blood cells with ACK lysing buffer (Thermo Fisher Scientific, MA, USA). BMDCs were plated in a 10 ml dish (1×10^6^ cells/mL) in RPMI 1640 (Thermo Fisher Scientific, MA, USA) supplemented with 10% heat-inactivated fetal bovine serum (FBS, Thermo Fisher Scientific, MA, USA), 100 U/mL penicillin (Thermo Fisher Scientific, MA, USA), 100 mg/mL streptomycin (Thermo Fisher Scientific, MA, USA), 25 μg/mL rmGM-CSF (Miltenyi Biotec, Bergisch Gladbach, GER), and 25 μg/mL rmIL-4 (Miltenyi Biotec, Bergisch Gladbach, GER) at 37°C in a humidified 5% CO_2_ atmosphere. On day 5 BMDCs were harvested, restimulated with new growth factors and plated at 1×10^6^ cells/mL on 24-well culture plate. BMDCs were treated with 25μM of quercetin from Sigma (Sigma-Aldrich, St Louis, MO, USA) on day 5 and day 7. On day 8 BMDCs were stimulated with 1 μg/mL of LPS (L6143, Sigma-Aldrich, St Louis, MO, USA) for 24 hours.

### Whole genome array

BMDCs were isolated and cultured as described. On day 5 and day 7 BMDCs were treated with ROBs-QP (25 μM). LPS was administered [1μg/ml] at day 8 and 6 hours later BMDCs were harvested. Total RNA was isolated with QIAzol (Qiagen, Hilden, GER) and treated with DNAase1 (Ambion). RNA integrity was assessed using the BioRad Experion System (BioRad Laboratories, CA, USA). RNA was amplified using the Illumina^®^ TotalPrep RNA Amplification kit (Ambion,). The quantity and quality of biotin-UTP incorporated cRNA was also assessed using the BioRad Experion System as previously described. Whole-Genome gene expression experiments were conducted using MouseRef-8 v2.0 Expression Bead-Chips (direct hybridization assay) on the Illumina iScan microarray platform (Illumina,). Data were processed through specific algorithms of filtration and cleaning of the signal of the Illumina Genome Studio Software (Cut off: Detection p-value <0.005; AVG signal < 100). Final output consisted of fluorescence intensity of each probe (AVG signal), representing the expression levels of each gene after quantile normalization. All the genes differentially expressed (“Differential Expression Analysis” with the “Illumina-custom error model” and with false Discovery Rate to adjust the P value) between groups were analyzed using the Core Analysis function of Ingenuity Pathway Analysis (Ingenuity System Inc, Redwood, CA) to identify biological functions, pathways and networks.

### qPCR analysis

Total RNA was isolated from BMDCs and human biopsies using TRIzol® (Thermo Fisher Scientific, MA, USA) according to manufacturer's instructions. 500 ng of total RNA was reverse transcribed with the High Capacity cDNA Reverse Transcription kit (Thermo Fisher Scientific, MA, USA) by using random primers for cDNA synthesis. Gene expression of Slpi and GAPDH was performed with TaqMan Gene Expression Assays (Thermo Fisher Scientific, MA, USA) - murine probes: Mm00441530_g1 and Mm00484668_m1, respectively; human probes: Hs00268204_m1 and Hs02758991_g1, respectively. Real-time analysis were run on CFX96 System (Biorad Laboratories, CA, USA) and the expression of all target genes was calculated relative to GAPDH expression using ΔΔCt method.

### ELISA

Cell culture supernatants were analyzed for TNFα release in triplicate, using an ELISA kit (R&D Systems, Minneapolis, MN, USA) following manufacturer' instructions.

### *In vivo* treatment of mice with quercetin

Mice were injected intragastrically with quercetin [0.5 μM/g] or vehicle at day 0, 3, 5 and 7. Starting from day 4, acute colitis was induced by administration of 2% DSS (MP Biomedicals, LLC, USA) in drinking water for 3 days. Mice were monitored on a daily basis for the following days after DSS administration. At day 7 mice were sacrificed, and the colon extracted, cut longitudinally and washed 3 times with cold 2.5mM EDTA to remove epithelial cells. The DCs/macrophages-enriched population was lysed with TRIzol® (Thermo Fisher Scientific, MA, USA) and used for total RNA extraction.

### *Ex vivo* treatment of human biopsies

Human intestinal biopsies from non-inflamed and inflamed areas of 7 patients with ileitis who underwent colonoscopy were analyzed 0013329 & 130702 (UTAS Animal Ethics Committee). For each patient an informed consent has been obtained. Human intestinal biopsies were collected into RPMI and treated with 25 μM of quercetin (Sigma-Aldrich, St Louis, MO, USA) for 4 hours in a humidified 5% CO_2_ atmosphere. After quercetin treatment, mRNA was extracted and Slpi expression was measured by qPCR as described.

### Immunohistochemistry (IHC) analysis

Immunohistochemistry (IHC) was performed on formalin fixed, paraffin embedded left colon biopsy tissue from healthy and ulcerative colitis patients. The sections were deparaffinized and subjected to 0.01 M citrate buffer (pH 6.0) antigen retrieval at 121 degrees for 4 mins in a decloaking chamber. Endogenous peroxidases were quenched by a 5 min incubation of 10% H_2_O_2_ in methanol. Non-specific binding was blocked by a 20 min incubation in Biocare background Sniper solution (BS966G, Biocare, Concord, CA21520, USA). Staining for human SLPI was performed using a specific antibody against human SLPI (PA5-20385, rabbit polyclonal, Thermo Fisher Scientific, Rockford, IL 61105, USA) at room temperature for 1 hour. Samples were then incubated with a horseradish peroxidase (HRP)-polymer (MRH53BL10, Biocare, Concord, CA21520, USA) for 30 mins, stained with Betazoid DAB chromogen (BDB900B, Biocare, Concord, CA21520, USA), counterstained with hematoxylin, dehydrated and mount in DPX.

### Small interfering RNA (siRNA)

siRNA transfection was performed in BMDCs culture from WT mice obtained as described before. Cells were cultured at 1×10^6^ in a 12-well plate and the transfection was carried out at day 4 using Lipofectamine 3000 (Thermo Fisher Scientific, MA, USA) in accordance with manufacturer's procedure. siRNA for Slpi were purchased from Thermo Fisher Scientific (s202008) and used at a final concentration of 40 pmol. In transfection experiments a mock-transfection control was performed by putting cells through the transfection procedure without adding siRNA. The Silencer Select Negative Control siRNA (4390843, Thermo Fisher Scientific, MA, USA) and Silencer® Select GAPDH Positive Control siRNA (4390849, Thermo Fisher Scientific, MA, USA) were used as negative control and positive control, respectively, for the setup of siRNA transfection. Each transfection experiment was done in triplicate. On day 5, cells were treated with 25 μM of quercetin and the day later cells were stimulated with 1 μg/ml of LPS. After 24 hours, cells were lysed with TRIzol® (Thermo Fisher Scientific, MA, USA) and used for total RNA extraction.

### Statistical analysis

All data were expressed as means ± SEM of data obtained from at least three independent experiments. We evaluated statistical significance with two-tailed Student's t test. Results were considered statistically significant at P < 0.05.
